# Dual role of icaritin in attenuating allograft rejection and exerting antitumor effects in mice

**DOI:** 10.3389/fimmu.2026.1762553

**Published:** 2026-03-18

**Authors:** Jinliang Duan, Shaofeng Chen, Yang Wang, Dejun Kong, Zhenglu Wang, Lei Cao, Wei Rao, Tao Chen, Sei Yoshida, Zhenzhou Wu, Hong Zheng, Zhongyang Shen

**Affiliations:** 1 Nankai University School of Medicine, Tianjin, China; 2 Tianjin Organ Transplantation Research Center, Tianjin, China; 3 Department of Pharmacy Intravenous Admixture Service, Maternal and Child Health Care Hospital of Weifang, Weifang, Shandong, China; 4 Biological Sample Resource Sharing Center, Tianjin First Central Hospital, Tianjin, China; 5 Organ Transplantation Center, the Affiliated Hospital of Qingdao University, Qingdao, Shandong, China; 6 Institute of Transplantation Medicine, Nankai University, Tianjin, China; 7 College of Life Sciences, Nankai University, Tianjin, China; 8 Tianjin Key Laboratory of Organ Transplantation, Tianjin First Central Hospital, Tianjin, China; 9 Key Laboratory of Transplant Medicine, Chinese Academy of Medical Science, Tianjin, China

**Keywords:** allograft rejection, hepatocellular carcinoma, icaritin, immunosuppressant, tumor recurrence

## Abstract

**Background:**

Long-term immunosuppression following organ transplantation results in an elevated risk of malignancies in recipients, which constitutes a major factor limiting their long-term survival. Therefore, the development of immunosuppressant with anti-tumor efficacy holds critical significance. Icaritin (ICT), a clinically employed antitumor drug, enhances anti tumor immunity by reshaping the tumor immune microenvironment. Moreover, recent evidence highlights its immunomodulatory role in mitigating multiple autoimmune diseases. However, whether ICT can attenuate the allograft rejection remains poorly characterized.

**Methods:**

Fully major histocompatibility complex-mismatched heterotopic heart transplantation was conducted from BALB/c mice to C57BL/6J mice. The rejection of the allografts was assessed via H&E staining and immunohistochemistry. Single-cell RNA sequencing (scRNA-seq) and flow cytometry were carried out on recipient splenocytes. In vitro, isolated naïve CD4^+^ T cells were cultured in Th1-polarizing conditioned medium with various treatments, and flow cytometry and quantitative PCR (qPCR) were employed to delineate the role of the Proviral integration site for Moloney murine leukemia virus (PIM1) during Th1 cell differentiation. Molecular docking, molecular dynamics simulations, and cellular thermal shift assay were employed to demonstrate the binding capacity between ICT and CCAAT/enhancer-binding protein β (CEBPB). A tumor-bearing murine heterotopic heart transplantation model was employed to demonstrate the dual efficacy of ICT in immunosuppression and antitumor.

**Results:**

ICT markedly attenuated acute cardiac allograft rejection and enhanced graft survival. scRNA-seq and flow cytometric analyses revealed a significant reduction in the proportion of splenic Th1 cells in ICT-treated recipient mice. *In vitro*, ICT suppressed CD4^+^ T-cell activation, proliferation, and Th1 cell differentiation in a dose-dependent manner. By binding to the transcription factor CEBPB, ICT inhibits PIM1 expression, and thereby suppresses the activation, proliferation, and Th1 differentiation of CD4^+^ T cells. In the tumor-bearing murine heart transplantation model, ICT potentiated the immunosuppressive efficacy of tacrolimus while reducing the tumor burden.

**Conclusions:**

While exerting antitumor effects, ICT attenuates allograft rejection by targeting the CEBPB/PIM1 axis, thereby suppressing CD4^+^ T-cell activation, proliferation, and Th1 differentiation.

## 1 Introduction

Organ transplantation is the optimal treatment for end-stage organ disease (1). Although the present immunosuppressants, such as calcineurin inhibitors are effective at attenuating rejection, long-term immunosuppressive exposure increases the risk of malignancy in recipients (2). Malignancy has become the second leading cause of death among transplant recipients (3). Therefore, exploring immunosuppressants with antitumor effects is of great importance. Rapamycin (RAPA) is an established immunosuppressant with antitumor properties that reduces incidence of malignancy in recipients (4). However, early postoperative administration increases the risk for hepatic artery thrombosis (HAT) and impaired wound healing (5). Thus, the need for immunosuppressive agents with antitumor activity persists.

Icaritin (ICT) is a flavone isolated from Epimedium brevicornum Maxim, that has a wide range of therapeutic potential such as antitumor effects, immune modulation and attenuation of ischemia–reperfusion injury (6–9). ICT reduces the infiltration of myeloid-derived suppressor cells in the tumor microenvironment and inhibits their expression of Programmed Cell Death Ligand 1 (PD-L1) (10). The combinatorial use of ICT and immune checkpoint inhibitors has been shown to achieve superior anti−tumor activity (11). A phase III clinical trial confirmed the safety and efficacy of ICT for the treatment of advanced hepatocellular carcinoma (HCC) (12). As a result, ICT has been approved by the Chinese National Medical Products Administration for use in unresectable HCC (13). In addition, ICT possesses immunomodulatory properties and has been reported to attenuate autoimmune diseases (14–16). However, whether ICT alleviates allograft rejection remains unclear.

CD4^+^ T cells play a key role in mediating allograft rejection (17). After transplantation, antigen-presenting cells (APCs) capture and process alloantigens, which are subsequently presented to naïve CD4^+^ T cells. This interaction triggers their activation, proliferation, and differentiation into various helper T-cell subsets. Among these, Th1 cells represent a predominant subtype involved in mediating allograft rejection. Th1 cells secrete proinflammatory cytokines such as interleukin 2 (IL-2), interferon-γ (IFN-γ) and tumor necrosis factor-α (TNF-α), which recruit and activate CD8^+^ cytotoxic T cells and macrophages, thereby promoting cell-mediated rejection. Fully mismatched allografts rejected by recipient mice display the Th1 cytokines IL-2 and IFN-γ together with anti-donor CD8^+^ cytotoxic T cells in the grafts (18), which implies that Th1 cells are indispensable in allogeneic transplant rejection.

Proviral integration site for Moloney murine leukemia virus 1 (PIM1) is a serine/threonine kinase that regulates several fundamental cellular processes, including cell survival, cycle progression, proliferation, and migration (19). Although PIM1 has been extensively studied in oncogenesis (20), recent research has increasingly highlighted its role in immunomodulation. In particular, PIM1 has been implicated in regulating CD4^+^ T-cell activation, proliferation, and differentiation, representing a promising therapeutic target for autoimmune diseases (21–23). CCAAT/enhancer-binding protein β (CEBPB), a member of the CCAAT/enhancer-binding protein family, functions as a b-Zip transcription factor involved in immune responses. Studies have demonstrated that CEBPB acts as a transcription factor for PIM1, directly binding to the PIM1 promoter and driving the expression of proinflammatory cytokines such as IL-1β and IL-6 (24). However, the role of the CEBPB/PIM1 axis in allograft rejection remains unexplored.

In this study, we established a murine allogeneic heart transplantation model to investigate the therapeutic effect of ICT in attenuating acute allograft rejection. To elucidate the impact of ICT on recipient immune cells, we constructed a comprehensive single-cell atlas using splenocytes isolated from recipient mice across different experimental groups, depicting alterations in immune cell composition and gene expression profiles induced by allogeneic transplantation and modified by ICT treatment. Our findings demonstrate that ICT attenuates acute allograft rejection primarily by inhibiting the CEBPB/PIM1 signaling axis, thereby suppressing CD4^+^ T-cell activation, proliferation, and Th1 cell differentiation. Furthermore, the tumor-bearing murine heterotopic heart transplant model was employed to demonstrate the dual efficacy of ICT in concurrently attenuating allograft rejection and exerting antitumor effects. These findings may support the clinical application of ICT as a promising immunosuppressant that decreases the risk for malignancy following transplantation.

## 2 Materials and methods

### 2.1 Chemical compounds and cell lines

ICT (MCE, HY-N0678) was dissolved in dimethyl sulfoxide for cell-based experiments or in a solvent composed of 85% normal saline and 15% Cremophor EL-40 (MCE, HY-Y1890) for animal studies.

Mouse Hepa1–6 HCC cells were obtained from the Chinese Academy of Sciences, China.

### 2.2 Animals

Male BALB/c (H2^d^) donor mice and C57BL/6J (H2^b^) recipient mice, aged 8–10 w and weighting 22–25 g, were purchased from Beijing Vital River Laboratory Animal Technology Co. Ltd. (Beijing, China). The mice were acclimatized for one week prior to the experiment under standard laboratory conditions with ad libitum access to autoclaved water and standard laboratory chow. All experiments were performed in accordance with the WMA Statement on animal use in biomedical research and approved by the Animal Ethics Committee of Genink Biotechnology Co., Ltd (Tianjin, China). The registration number of the ethical approval is GENINK-20240039.

### 2.3 Heterotopic heart transplantation and animal groups

The mice were randomly assigned to the various groups. Hearts from BALB/c donors were transplanted to C57BL/6J recipients, the procedure was conducted as previously described (25). Briefly, donors were anesthetized via 2% isoflurane and administered via a nose cone mask. Donor hearts were harvested and subsequently implanted into the peritoneal cavity of recipient mice. Vascular anastomoses were established by connecting the donor’s aorta to the recipient’s abdominal aorta and the donor’s pulmonary artery to the recipient’s inferior vena cava. After being resuscitated, the recipient mice were administered ICT, tacrolimus or an equivalent volume of solvent by gavage twice daily. After 7 d of intervention, the recipients were euthanized, followed by cervical dislocation to obtain tissue samples. The abbreviations used to represent these groups were as follows: control (CON), allotransplantation (ALLO), allotransplantation with 40 mg/kg/d ICT treatment (ICT), and allotransplantation with 1 mg/kg/d tacrolimus treatment (FK506).

### 2.4 Histopathology and immunohistochemistry analyses

The harvested cardiac allografts were fixed in 10% formalin, paraffin-embedded, and sectioned into 5-μm slices for H&E staining. The severity of allograft rejection, including lymphocyte infiltration, vasculitis, infarction, myocyte necrosis, intravascular thrombosis, and interstitial hemorrhage, was evaluated on the basis of established criteria (26). Specifically, pathological alterations were graded on a 0–4 scale relative to normal tissue: 0 (none), 1 (minimal), 2 (mild), 3 (moderate), and 4 (marked).

To assess the infiltration of CD4^+^ and CD8^+^ cells in the different groups, immunohistochemistry (IHC) was performed as previously described. Briefly, the slices were incubated at 65 °C for 4 h, after which One-step Dewaxing/Antigen Retrieval Buffer (Elabscience, E-IR-R220A) was used. After antigen retrieval with Tris-EDTA (pH=9) and blocking with 10% goat serum, the tissue sections were incubated overnight at 4 °C with anti-mouse CD4 antibody (1:1000, Abcam ab183685) and anti-CD8 antibody (1:1000, Abcam ab217344). After incubation with an HRP-conjugated secondary antibody, image acquisition and analysis were performed via ImageJ software.

### 2.5 Multiple cytokines assay

Serum concentrations of IL-1β, IL-2, IL-4, IL-6, IL-10, IL-12p70, IL-17a, IFN-γ, and TNF-α were quantified with the ABplex Mouse 9-Plex Custom Panel (ABclonal, RK04383) following the manufacturer’s protocol. In brief, the samples were incubated with antibody-conjugated beads and subsequently analyzed by flow cytometry.

### 2.6 Tissue dissociation and preparation for single-cell suspensions

The spleens harvested from the recipient mice of CON (n=2), ALLO (n=3) and ICT (n=3) groups were cut into 0.5 mm^2^ pieces and washed with phosphate solution (PBS), and nonpurpose tissues were removed. Then the tissues were dissociated into single cells in dissociation solution (0.35% collagenase IV5, 2 mg/ml papain, 120 units/ml DNase I) in a 37 °C water bath with shaking for 20 min. After termination of digestion with PBS containing 10% fetal bovine serum (FBS), the resulting cell suspension was filtered through a 70um stacked cell strainer and centrifuged. Next, the cell pellet was treated with ACK lysing Buffer (Gibco, A10492-01) to lyse the remaining red blood cells. Then, Miltenyi ^®^ Dead Cell Removal Kit (Miltenyi,130-090-101) was used to remove dead cells. Finally, overall cell viability was confirmed by trypan blue exclusion, which was required to be > 85%. Single–cell suspensions were counted via a haemocytometer, and the concentration was adjusted to 700–1200 cells/μL.

### 2.7 Chromium 10x genomics library and sequencing

Single-cell suspensions were processed via the 10x Genomics Chromium Single-Cell 3’ Kit (V3) on the 10x Chromium platform to capture approximately 10,000 cells, following the manufacturer’s protocol. Subsequent cDNA amplification and library preparation were performed according to standard procedures. LC-Bio Technology Co., Ltd. (Hangzhou, China) performed paired-end (150 bp) sequencing on an Illumina NovaSeq 6000 system, achieving a minimum depth of 20,000 reads per cell.

### 2.8 Bioinformatics analysis

The raw scRNA-seq data were demultiplexing and FASTQ conversion via Illumina bcl2fastq (v2.20). Subsequently, Cell Ranger was employed to conduct data quality statistics. The data were then aligned to Mus_musculus.GRCm39.Ensembl/v105 reference genome.

The output from Cell Ranger was imported into Seurat (version 4.1.0) for dimensionality reduction, clustering, and analysis. Quality control filtering utilized the following thresholds: minimum gene detection in 3 cells, >500 genes expressed per cell, and <25% mitochondrial gene content per cell. For data visualization, we applied Seurat to reduce the dimensionality of all the cells and used Uniform Manifold Approximation and Projection (UMAP) to project them into a two-dimensional space. For cell cluster identification and marker detection, a weighted Shared Nearest Neighbor graph-based clustering approach was implemented. Then, the marker genes for each cell cluster were identified using the default parameter “bimod” via the FindAllMarkers function in Seurat. Specifically, genes that were expressed in more than 10% of the cells in a cluster and had an average log (fold change) greater than 0.26 were selected as marker genes. For the identification of differentially expressed genes (DEGs) between two groups of the same cell types, the default parameters of the FindMarkers function in Seurat were used. After the genes with a minimum log_2_ (fold change) of 0.26 and a maximum adjusted p - value of 0.01 were processed, they were categorized by average log_2_ (fold change). Gene Ontology (GO) terms and Kyoto Encyclopedia of Genes and Genomes (KEGG) pathways were used to create gene sets, which serve as a basis for further functional analysis. Finally, Gene Set Enrichment Analysis (GSEA, version 4.2.3) was conducted to identify the pathway enrichment of all genes between the two groups of the same cell type. The screening thresholds were set as |NES| > 1, NOM.pval < 0.05, and FDR.qval < 0.25.

### 2.9 Flow cytometry

Splenocytes and CD4^+^ T cells cultured *in vitro* were harvested for analysis. A Zombie Aqua™ Fixable Viability Kit (Biolegend, 423101) was used according to the manufacturer’s instructions to rule out dead cells. Following Fc receptor blockade with TruStain FcX™ PLUS (BioLegend, 156603), cell surface staining was performed with fluorochrome-conjugated antibodies against CD3, CD4, CD8, CD19, CD11c, F4/80, and NK1.1. Following fixation and permeabilization, the cells were stained intracellularly with fluorochrome-conjugated antibodies for detection of IFN-γ, IL-4, IL-17A, Foxp3 and PIM1.

For intracellular cytokine staining, the cells were stimulated with Cell Stimulation Cocktail (plus protein transport inhibitors, Invitrogen, 00-4975-03) according to the manufacturer’s instructions for 5–12 h before staining.

### 2.10 *In vitro* cell culture

Naïve CD4^+^ T cells were isolated from the spleens of C57BL/6J mice via the EasySep™ Mouse Naïve CD4^+^ T Cell Isolation Kit (Stemcell Technologies, 19765) and identified by flow cytometry. The isolated naïve CD4^+^ T cells were cultured in 96-well plates pre-coated with an anti-mouse CD3 functional antibody (5 μg/mL, Biolegend, 100201) in Th1-polarizing conditioned medium (anti-mouse CD28 antibody: 2 μg/mL, Biolegend, 102116; IL-2: 20 ng/mL, Biolegend,575402; IL-12p70: 10 ng/mL, Peprotech, 210-12; anti-mouse IL-4: 10 μg/mL, Biolegend, 504122);. ICT or vehicle (0.1% DMSO) was added at the indicated concentrations. After 72 h, the cells from all the groups were collected for flow cytometry analysis.

The Hepa1–6 cell line was cultured in Dulbecco’s Modified Eagle’s Medium supplemented with 10% FBS and 1% penicillin–streptomycin for use in the *in vivo* tumor model.

### 2.11 CCK-8 assay

To assess the dose-dependent effects of ICT on cellular activity, the cells were cultured for 72 h, after which 10 μL of CCK-8 reagent (GLPBIO, GK10001) was added to each well. After 2 h of incubation, the absorbance at 450 nm was measured via a microplate reader to determine cell viability.

### 2.12 Mixed lymphocyte reaction

A mixed lymphocyte reaction (MLR) was conducted to evaluate the effects of ICT on the alloreactive proliferation of immune cells. Briefly, splenocytes from BALB/c mice and C57BL/6J mice were cocultured at a 1:1 ratio for 72 h. ICT was added at the indicated concentrations upon culture initiation. Lymphocyte proliferation was quantified via a CCK-8 assay (GLPBIO, GK10001) by measuring the absorbance at 450 nm after 2 h of incubation.

### 2.13 Lymphocyte cell proliferation assay

CFSE (Thermo Fisher, C34554) was used to assess the proliferative capacity of CD4^+^ T cells according to the manufacturer’s protocol. Briefly, PBS was used to wash the isolated CD4^+^ T cells to remove any serum. Next, the cells were incubated with 3 µM CFSE working solution for 10 min at room temperature. Then, cold complete media (containing 10% FBS) was added to stop labeling. After being cultured in a 96-well plate for 72 h, the cells were collected for flow cytometry analysis to evaluate the cell proliferation rate.

### 2.14 Quantitative real-time PCR

Total RNA from CD4^+^ T cells was extracted with TRIzol (Thermo Fisher, 15596018CN) and used to synthesize cDNA (Vazyme, R323). qPCR was performed using ChamQ Blue Universal SYBR qPCR Master Mix (Vazyme,Q312). The relative mRNA expression of PIM1 was determined via normalization of the expression of each target gene to that of β-actin via the 2^–ΔΔCt^ method. The primer sequences for PIM1 were as follows:

forward: 5′- CTGGAGTCGCAGTACCAGG-3′,

reverse: 5′- CAGTTCTCCCCAATCGGAAATC-3′.

### 2.15 Molecular docking and molecular dynamics simulation

The 3D structure of CEBPB (PDB ID: 2E42) was retrieved from the Protein Data Bank in Europe (https://www.ebi.ac.uk/pdbe/entry/pdb/2e42). The structure of ICT (PubChem CID: 5318980) was obtained from PubChem (https://pubchem.ncbi.nlm.nih.gov/compound/5318980). Molecular docking of the small molecule to the target protein was performed via AutoDock Vina. The results were analyzed using the PLIP (Protein-Ligand Interaction Profiler) system. The highest-scoring docking poses were visualized and assessed using PyMOL.

Molecular dynamics simulations of the protein–ligand complex were performed via the GROMACS 2025.2 software package. The system was placed in a periodic boundary cubic box and solvated with the TIP3P water model under the CHARMM36 force field, and ions were added to achieve a physiological concentration (150 mM NaCl). Following energy minimization and stepwise NVT/NPT equilibration under positional restraints (100 ps each), a production MD run was conducted for 100 ns with an integration time step of 2 fs. The trajectories were processed to remove periodic artifacts and recenter the system. Analyses included the root mean square deviation (RMSD) and radius of gyration (Rg) of protein backbone Cα atoms, residue–wise root mean square fluctuation (RMSF), solvent accessible surface area (SASA), and number of hydrogen bonds between the protein and ligand. Furthermore, 2D and 3D free energy landscape (FEL) plots were constructed to visualize the conformational stability of the complex.

### 2.16 Cellular thermal shift assay

CD4^+^ T cells were treated with ICT (4 μM) or DMSO for 72 h. Cells from each group were harvested, washed, and resuspended in ice-cold PBS containing a 1% protease inhibitor cocktail. The suspensions were evenly aliquoted into 5 PCR tubes and incubated at designated temperatures for 3 min. Following two freeze–thaw cycles in liquid nitrogen, the lysates were centrifuged at 20,000 × g for 20 min and the supernatants were collected. Equal amounts of protein from each sample were separated by SDS-PAGE, transferred to PVDF membranes, and probed overnight at 4°C with an anti-CEBPB primary antibody (Abcam, ab32358). After incubation with an HRP-conjugated secondary antibody, signals were detected using enhanced chemiluminescence and quantified by ImageJ.

### 2.17 HCC-bearing murine heterotopic heart transplant model

Hepa1–6 cells were collected, washed with PBS, and resuspended at a concentration of 1×10^7^ cells/ml. A 100 μL aliquot of the cell suspension was subcutaneously injected into the right flank of C57BL/6 mice. After 7 d, these HCC-bearing mice served as recipients for heterotopic heart transplantation from BALB/c donors. On day 14, the spleens were collected from the recipient mice for immune response assessment, the cardiac grafts were collected for rejection evaluation, and the tumors were weighed.

### 2.18 Statistics

Statistical analyses were performed via GraphPad Prism 9.0. The data are expressed as mean ± SDs. Intergroup comparisons were conducted as follows: one-way ANOVA for multigroup analyses, and unpaired Student’s t - test for two-group comparisons. Graft survival curves were generated by the Kaplan–Meier method, with statistical significance assessed via the log-rank (Mantel-Cox) test. *P*-values > 0.05 were deemed statistically insignificant, and were denoted as NS. Significance levels were indicated as follows: *P < 0.05, **P < 0.01, ***P < 0.001, ****P < 0.0001.

## 3 Results

### 3.1 ICT treatment attenuates cardiac allograft rejection in mice

**Figure 1:**
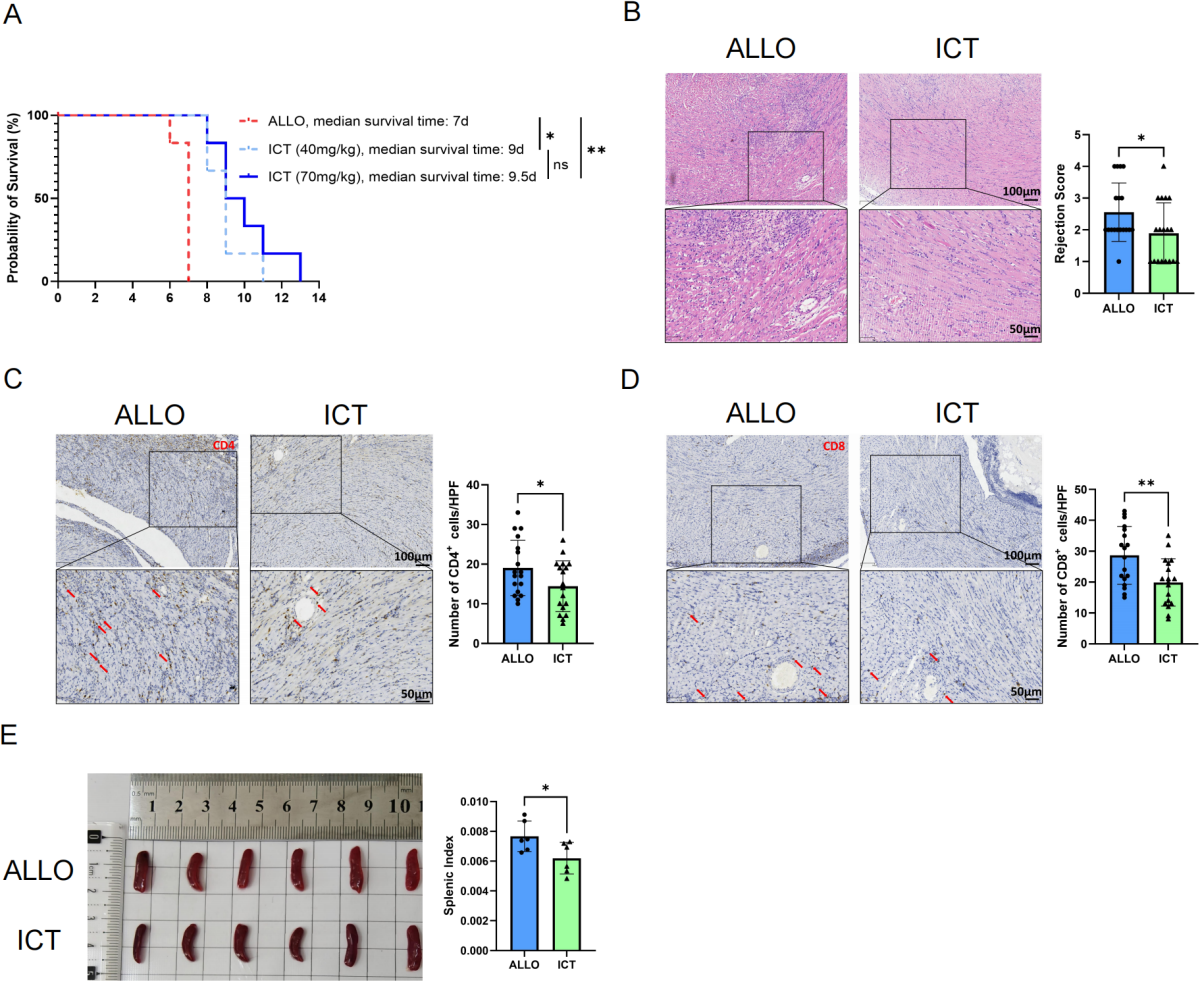
ICT attenuates acute rejection and prolongs survival of cardiac allografts in mice. **(A)** Kaplan - Meier curves of cardiac allograft survival. **(B-E)** Allografts were harvested on day 7 post -transplant. **(B)** Allograft histopathological changes were evaluated by H&E staining across groups. Acute rejection severity was graded based on cardiac rejection scoring criteria, with 3 randomly selected areas analyzed per slide. CD4^+^ cell **(C)** and CD8^+^ cell **(D)** infiltration in allografts was detected by immunohistochemical staining. Positive cells (indicated by arrows) were quantified across 3 randomly selected areas per slide. **(E)** The spleen indices of the recipients. Statistical analysis was conducted by Log - rank test **(A)** and Student t-test **(B-E)** (n = 6 mice per group). Data are shown as mean ± SD; ns, *p* > 0.05, *p < 0.05, **p < 0.01.

To assess the therapeutic effect of ICT in attenuating acute allograft rejection, fully major histocompatibility complex-mismatched heterotopic heart transplantation was conducted from BALB/c mice to C57BL/6J mice. Following transplantation, the recipients were administered ICT at two antitumor doses [low-dose: 40 mg/kg/d (27); high-dose: 70 mg/kg/d (10)] or an equivalent volume of solvent by gavage. As shown in **Figure 1A**, ICT treatment at 40 mg/kg/d significantly prolonged the graft survival time from 7 d in control mice to 9 d, and ICT treatment at 70 mg/kg/d resulted in additional extension of the graft survival time to 9.5 d. On day 7 after transplantation, cardiac allografts harvested from the ALLO group recipient mice exhibited pronounced infiltration of inflammatory cells, accompanied by hemorrhage and significant disruption of the cardiac architecture. Notably, low-dose ICT treatment notably reduced allograft damage and rejection severity, as evidenced by a lower rejection score (**Figure 1B**). Subsequently, we assessed the intragraft infiltration of CD4^+^ and CD8^+^ cells by IHC staining, which showed that low-dose ICT treatment reduced the infiltration of CD4^+^ (**Figure 1C**) and CD8^+^ (**Figure 1D**) cells in the grafts. The weight of the spleen and the splenic index (**Figure 1E**) in the ICT group were significantly lower than in the ALLO group, suggesting that ICT could inhibit alloantigen-driven splenocyte proliferation. Collectively, these findings indicate that ICT effectively attenuates allograft rejection following transplantation.

### 3.2 scRNA-seq-based immune cell landscape in the recipients’ spleens

**Figure 2:**
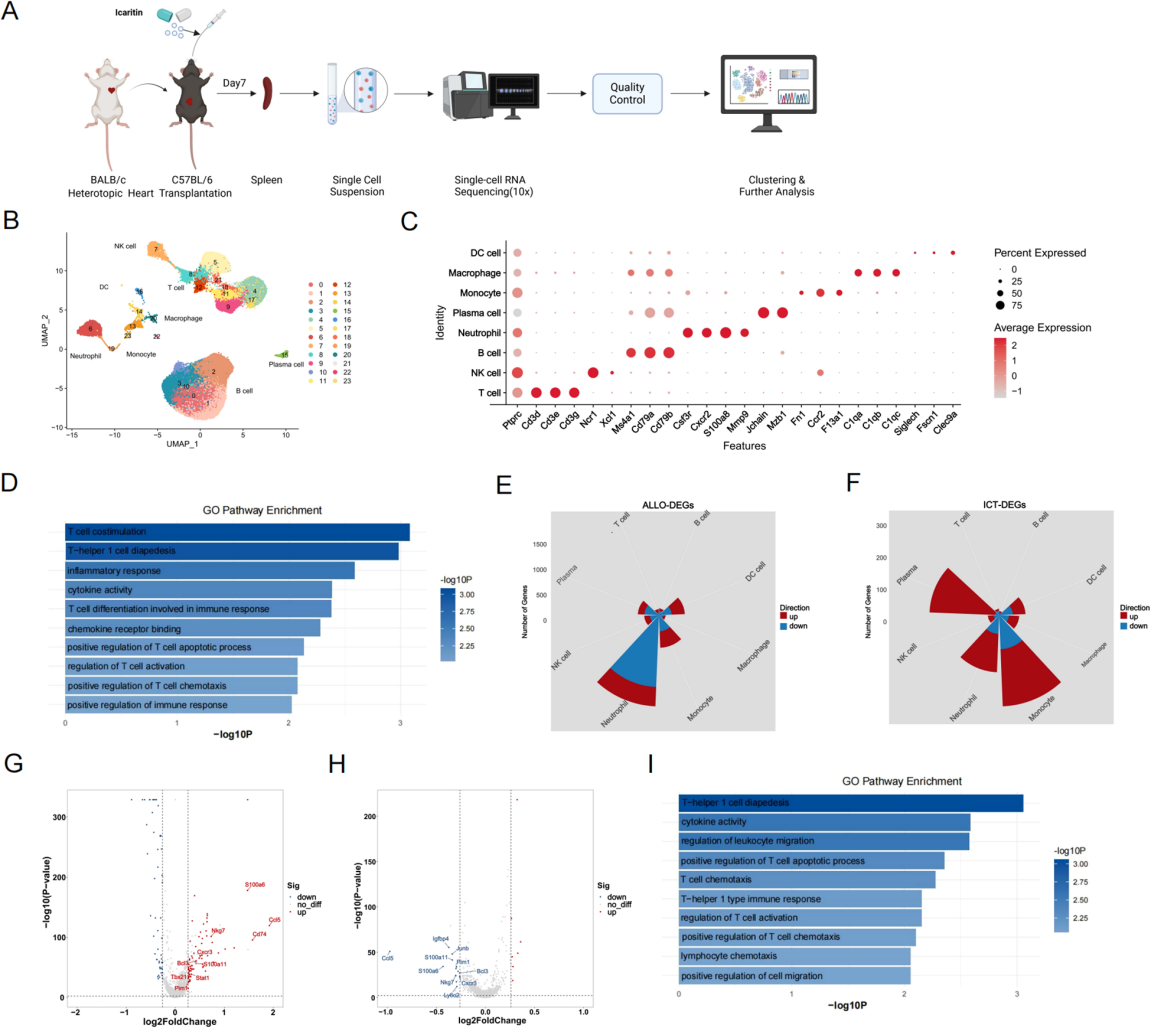
scRNA-seq analysis reveals immune cell alterations in the spleen of recipient mice. **(A)** Schematic illustrating the establishment of murine heart transplantation model and the sample processing workflow for scRNA-seq. **(B)** UMAP plot depicting 88,516 total cells, clustered into 23 color-coded subclusters and 8 major cell types. **(C)** Dot plot illustrating column-wise Z-score expression (color) and percentage of positive cells (dot size) for selected genes (rows) among 8 cell populations (columns). **(D)** GO terms enriched by downregulated ICT-DEGs in all cells. **(E)** Rose diagrams quantifying up/down-regulated ALLO-DEGs among all immune cell subsets. **(F)** Rose diagrams quantifying up/down-regulated ICT-DEGs among all immune cell subsets. **(G)** Volcano plot showing the upregulated ALLO-DEGs in T cells. **(H)** Volcano plot showing the downregulated ICT-DEGs in T cells. **(I)** GO terms enriched in downregulated ICT-DEGs in T cells.

To explore the immunoregulatory effect of ICT on immune cells, recipient spleens were collected from the CON, ALLO, and ICT groups for scRNA-seq analysis (**Figure 2A**). The analysis included 88,516 high-quality cells, which were categorized into 23 subclusters and 8 main cell types, identified on the basis of typical marker genes and the top 10 enriched genes (**Figures 2B, C**). The marker genes for the 8 main cell types are as follows: *Cd3d*, *Cd3e*, and *Cd3g* for T cells; *Ncr1* and *Xcl1* for NK cells; *Ms4a1*, *Cd79a*, and *Cd79b* for B cells; *Csf3r*, *Cxcr2*, and *S100a8* for neutrophils; *Jchain* and *Mzb* for plasma cells; *Fn1*, *Ccr2*, and *F13a1* for monocytes; *C1qa*, *C1qb*, and *C1qc* for macrophages; and *Siglech*, *Fscn1*, and *Clec9a* for dendritic cells (**Figure 2C**). DEGs between the ALLO group and CON group were designated ALLO-DEGs, whereas those between the ICT-treated group and ALLO group were designated ICT-DEGs. GO analysis showed that the downregulated ICT-DEGs were enriched in T-helper 1 cell diapedesis, inflammatory response, T cell differentiation involved in immune response, regulation of T cell activation and positive regulation of T cell chemotaxis (**Figure 2D**), suggesting that ICT attenuates allograft rejection potentially through inhibiting the activation, proliferation and migration of T cells. To further investigate the effects of allotransplantation and ICT in a cell type-specific manner, we analyzed the ALLO-DEGs and ICT-DEGs in each cell type. Neutrophils and monocyte cells exhibited more DEGs than other cell types did after allotransplantation and ICT treatment (**Figures 2E, F**). We investigated ALLO-DEGs and ICT-DEGs in T cells as they play key roles in allograft rejection. As shown in **Figure 2G**, *Ccl5*, *Nkg7*, and *Cxcr3* were significantly upregulated in the ALLO group compared with the CON group, while these genes were downregulated between the ICT group and ALLO group (**Figure 2H**), suggesting that ICT may inhibit the cytotoxicity and migratory capacity of T cells during allogeneic stimulation. In addition, GO analysis showed that the downregulated ICT-DEGs in T cells were enriched in T-helper 1 cell diapedesis, T cell chemotaxis, T-helper 1 type immune response and regulation of T cell activation (**Figure 2I**). To summarize, ICT remodeled the gene signatures of immune cells, particularly T cells, following allogeneic transplantation.

### 3.3 scRNA-seq analysis reveals altered gene expression in T–cell subsets

Previous studies have shown that the T–cell response to alloantigens is crucial in determining the outcomes of solid organ transplantation (28). Therefore, we investigated the altered gene expression in T cell subsets and their functional characteristics during acute allograft rejection.

**Figure 3:**
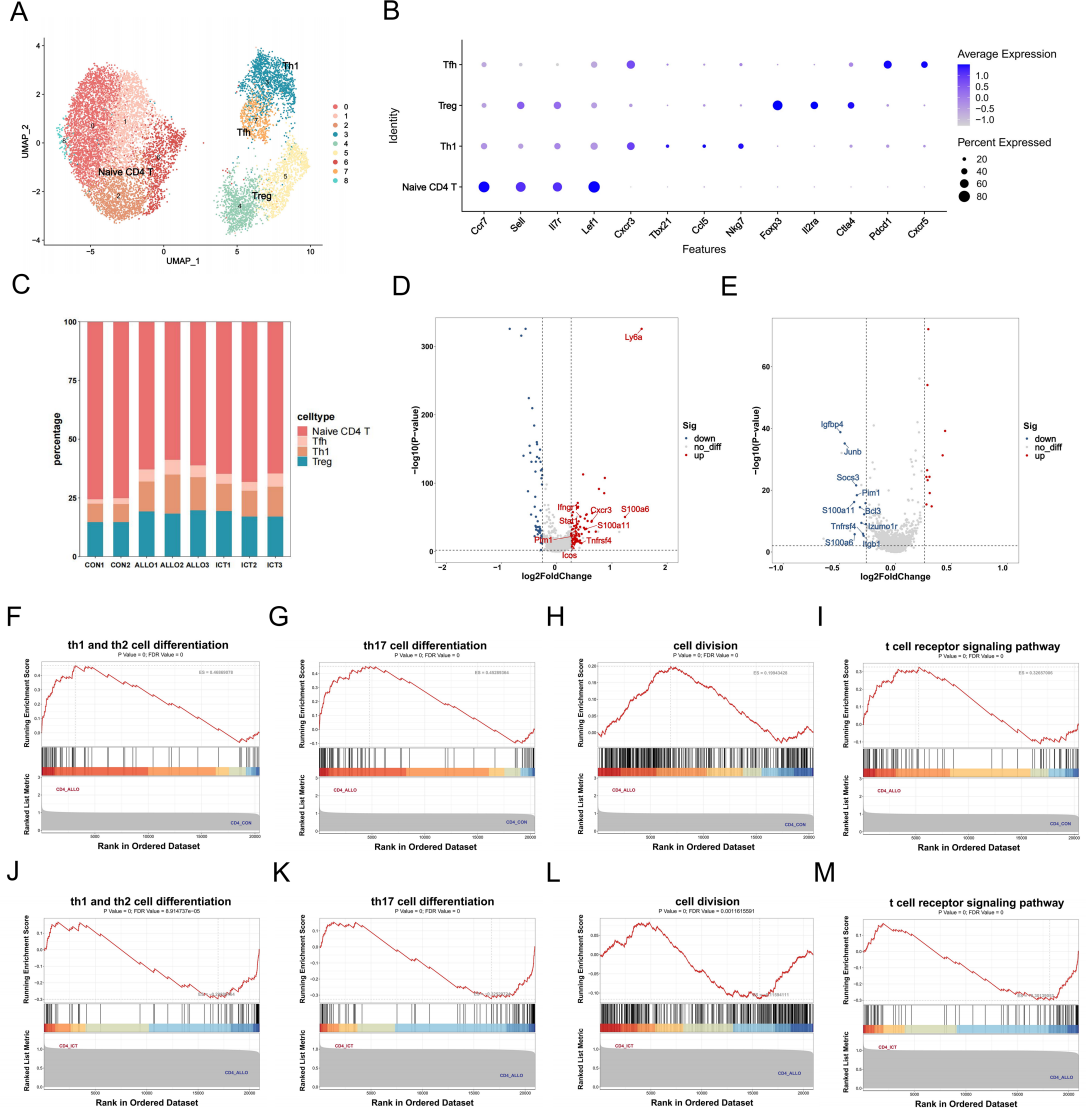
scRNA-seq analysis of the ICT mediated response in CD4^+^ T cells. **(A)** UMAP plots displaying 9 color-coded subclusters of CD4^+^ T cells. **(B)** Dot plot illustrating column-wise Z-score expression (color) and percentage of positive cells (dot size) for selected genes (rows) among the 4 cell types (columns). **(C)** Bar chart showing the proportional distribution of CD4^+^ T cell subtypes. **(D)** Volcano plot showing the ALLO-DEGs in CD4^+^ T cells. **(E)** Volcano plot showing the ICT-DEGs in CD4^+^ T cells. **(F-I)** GSEA analysis of upregulated ALLO-DEGs enriched in KEGG in CD4^+^ T cells. **(J-M)** GSEA analysis of downregulated ICT-DEGs enriched in KEGG in CD4^+^ T cells.

As shown in **Figures 3A, B**, CD4^+^ T cells were reclustered into 9 subsets and 4 main cell types. The marker genes for the 4 main cell types are as follows: *Ccr7*, *Sell*, *Il7r* and *Lef1* for naïve CD4^+^ T cells; *Cxcr3*, *Tbx21*, *Ccl5* and *Nkg7* for Th1 cells; *Foxp3*, *Il2ra* and *Clta4* for Tregs; and *Pdcd1* and *Cxcr5* for Tfh cells. As shown in **Figure 3C**, Th1 cells, key participants in rejection, were significantly increased in the ALLO group compared with those in the CON group, while ICT treatment significantly decreased the percentage of Th1 cells after transplantation.

To further investigate the impact of ICT on CD4^+^ T cells following allogeneic transplantation at the molecular level, we analyzed the DEGs in CD4^+^ T cells between the ALLO group and CON group, as well as the DEGs between the ICT group and ALLO group. As shown in **Figures 3D**, allotransplantation upregulated Th1 cell-related signature genes (*Cxcr3*, *Stat1*) and costimulation-related signature genes (*Tnfrsf4*, *Icos*). Furthermore, the changes in *Tnfrsf4* expression were reversed by ICT (**Figure 3E**). GSEA analysis showed that Th1 and Th2 differentiation signaling (**Figure 3F**), Th17 differentiation signaling (**Figure 3G**), cell division signaling (**Figure 3H**), and T cell receptor signaling pathway (**Figure 3I**) were upregulated in the ALLO group, and downregulated by ICT treatment (**Figures 3J–M**).

CD8^+^ T cells were reclustered into 3 main cell types (**Supplementary Figure 1A**). As expected, the proportion of cytotoxic T lymphocytes (CTLs) in the ALLO group was significantly higher than those in the CON group, while it was decreased by ICT treatment (**Supplementary Figure 1B**). Furthermore, we analyzed the upregulated ALLO-DEGs and downregulated ICT-DEGs in CD8^+^ T cells, which showed that the expression of cytotoxic genes such as *Gzma*, *Gzmm*, and *Nkg7* was significantly upregulated in the ALLO group (**Supplementary Figure 1C**), while it was downregulated following ICT treatment (**Supplementary Figure 1D**). GSEA analysis showed that the cell division signaling pathway was upregulated in the ALLO group (**Supplementary Figure 1E**) and downregulated by ICT treatment (**Supplementary Figure 1F**). Additionally, we calculated scores for the regulation of T cell migration pathway, and the results suggested that ICT can reduce the migratory ability of CD8^+^ T cells after transplantation (**Supplementary Figure 1G**). These results suggest that ICT inhibits the response of T cells to allografts.

### 3.4 ICT inhibits CD4^+^ T-cell proliferation and Th1 cell differentiation in the spleen after cardiac transplantation

**Figure 4:**
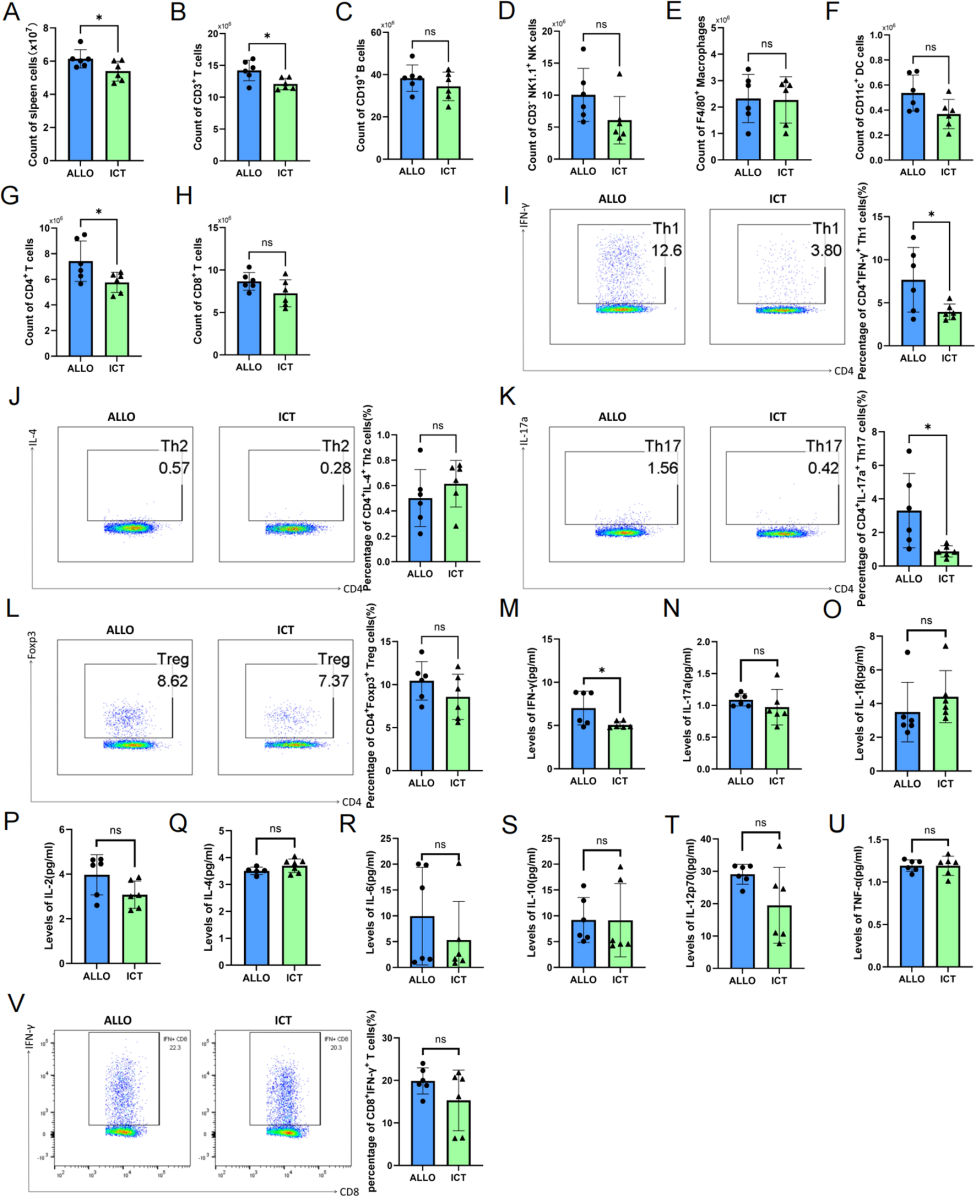
ICT reduces CD4^+^ T-cell count and Th1 cell proportion in recipient spleens. Bar graph showing the count of total spleen cells **(A)**, CD3^+^ T cells **(B)**, CD19^+^ B cells **(C)**, CD3^-^ NK1.1^+^ NK cells **(D)**, F4/80^+^ macrophages **(E)**, CD11c^+^ dendritic cells **(F)**, CD4^+^ T cells **(G)**, CD8^+^ T cells **(H)**. Flow cytometry analysis of the percentage of CD4^+^ IFN-γ^+^ Th1 cells **(I)**, CD4^+^ IL-4^+^ Th2 cells **(J)**, CD4^+^ IL-17a^+^ Th17 cells **(K)**, CD4^+^ Foxp3^+^ Treg cells **(L-U)** Peripheral levels of IFN-γ, TNF-α, IL-1β, IL-2, IL-4, IL-6, IL-10, IL-12p70 and IL-17a. **(V)** Flow cytometry analysis of the percentage of CD8^+^ IFN-γ^+^ cells. Statistical analysis between two groups was conducted by Student’s t-test (n = 6 mice per group). Data are shown as mean ± SD; ns, *p* > 0.05, *p < 0.05.

To validate the effects of ICT on immune cells based on scRNA-seq analysis, we examined total cell counts in the spleens of recipients and quantified the proportions of various immune cell types, including T cells, B cells, NK cells, dendritic cells, and macrophages (**Supplementary Figure 2A**). As shown in **Figures 4A**, ICT significantly reduced the total number of cells in the spleen. Although no substantial changes were observed in the proportions of the various immune cell types (**Supplementary Figures 2B–E**), the cell count of T cells in the ICT group was markedly lower than that in the ALLO group (**Figure 4B**). There were no significant differences in the counts of B cells, NK cells, dendritic cells, or macrophages (**Figures 4C–F**). Further analysis revealed that ICT treatment specifically decreased the total number of CD4^+^ T cells (**Figure 4G**), whereas the count of CD8^+^ T cells showed a slight decrease without statistical significance (**Figure 4H**). Additionally, CD4^+^ T cell subsets were analyzed in more details (**Supplementary Figure 2F**). Compared with the ALLO group, ICT treatment significantly reduced the percentages of CD4^+^IFNγ^+^ Th1 cells (**Figure 4I**) and CD4^+^IL-17a^+^ Th17 cells (**Figure 4K**) in the spleen, while no notable effects were observed on the proportions of CD4^+^IL-4^+^ Th2 cells (**Figure 4J**) or CD4^+^ Foxp3^+^ Treg cells (**Figure 4L**). Serum levels of inflammatory cytokines were also measured. ICT treatment significantly decreased IFN-γ levels (**Figure 4M**), with no significant changes observed for IL-17a, IL-1β, IL-2, IL-4, IL-6, IL-10, IL-12p70, or TNF-α (**Figures 4N–U**). Moreover, no appreciable changes were detected in the proportions of IFN-γ^+^ CD8^+^ T cells (**Figure 4V**). Taken together, these results suggest that ICT inhibits the differentiation of CD4^+^ T cells into Th1 subsets in the spleens of recipients.

### 3.5 ICT inhibits CD4^+^ T-cell activation, proliferation and Th1 cell differentiation *in vitro*

**Figure 5:**
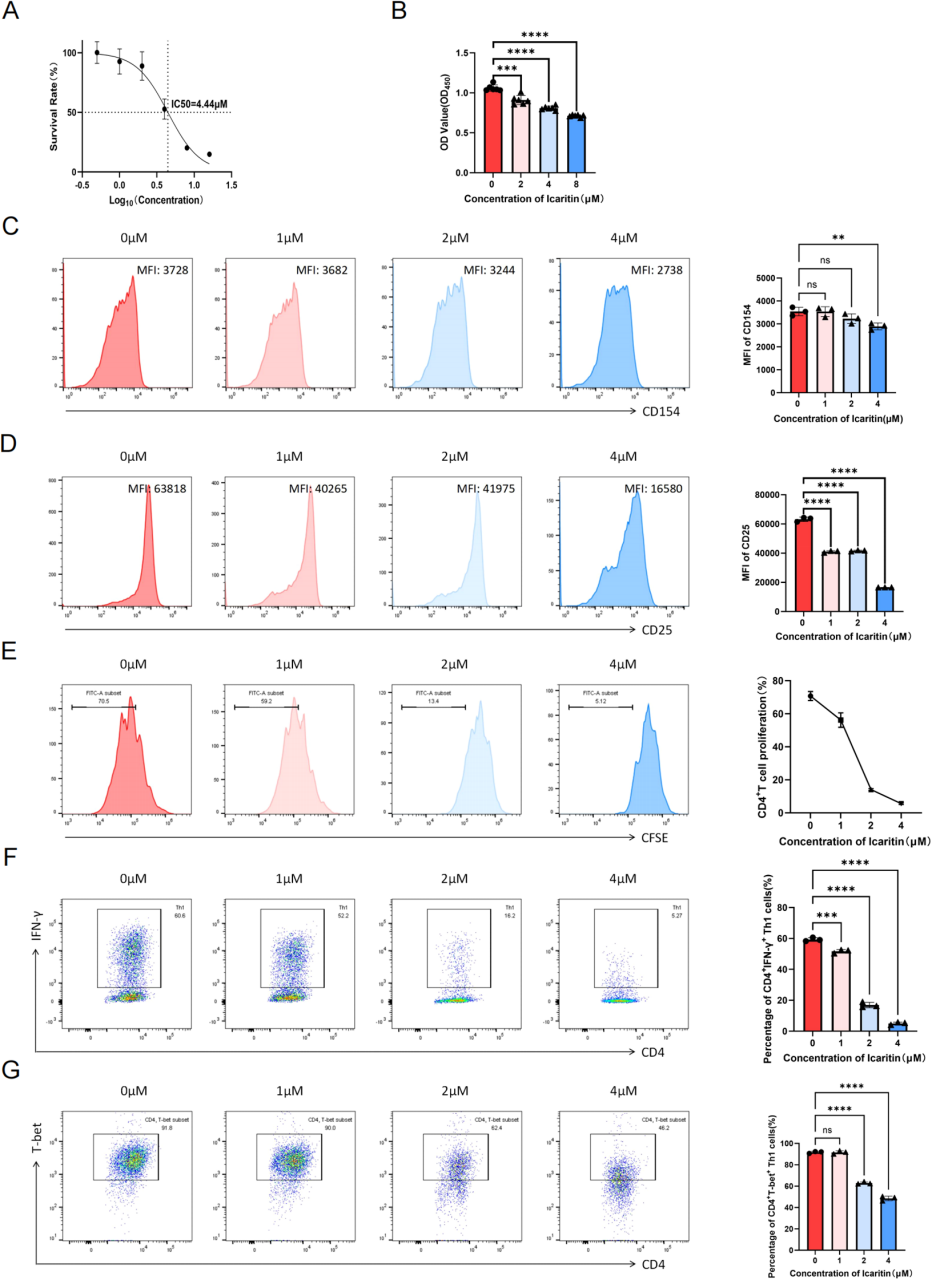
ICT inhibits CD4^+^ T-cell activation, proliferation and Th1 cell differentiation in a dose-dependent manner. **(A)** IC_50_ of ICT on CD4^+^ T cells. **(B)** Proliferation of spleen cells detected by CCK-8. Flow cytometry analysis of the CD154 **(C)** and CD25 **(D)** expression levels in CD4^+^ T cells treated with different concentration of ICT. **(E)** Proliferation of CD4^+^ T cells treated with different concentrations of ICT. **(F, G)** Flow cytometry analysis of the percentages of CD4^+^ IFN-γ^+^ Th1 and CD4^+^ T-bet^+^ cells treated with different concentrations of ICT. Statistical analysis was conducted by one-way ANOVA (n = 3 per group). Data are shown as mean ± SD; ns, *p* > 0.05, **p < 0.01, ***p < 0.001, ****p < 0.0001.

Given that ICT reduces the total number of CD4^+^ T cells and the proportion of Th1 cells in recipients’ spleens, we next investigated whether ICT directly regulates CD4^+^ T cells *in vitro*. Using the CCK-8 assay, the IC_50_ of ICT for CD4^+^ T cells was determined to be 4.4 µM (**Figure 5A**). Subsequently, a concentration gradient within the range of the IC_50_ was selected for the following *in vitro* experiments. As shown in **Figure 5B**, ICT effectively suppressed the mixed lymphocyte reaction in a dose-dependent manner. Next, we measured the expression of the T-cell activation marker CD154 at 6 h and CD25 at 72 h following CD4^+^ T-cell activation. The results demonstrated that ICT effectively inhibited CD4^+^ T-cell activation (**Figures 5C, D**). Furthermore, CFSE labeling was employed to evaluate the inhibitory effect of ICT on the proliferative capacity of CD4^+^ T cells. As shown in **Figures 5E**, ICT effectively suppressed CD4^+^ T cell proliferation in a dose-dependent manner. We incorporated ICT into the Th1 cell differentiation-conditioned medium to investigate its effects on Th1 cell differentiation, which indicated that ICT significantly inhibited Th1 cell differentiation by diminishing the expression of IFN-γ (**Figure 5F**, **Supplementary Figure 3A**) and T-bet (**Figure 5G**, **Supplementary Figure 3B**). In summary, these findings reveal that ICT directly inhibits CD4^+^ T-cell activation, proliferation and Th1 cell differentiation *in vitro*.

### 3.6 Identification of PIM1 as a target of ICT in CD4^+^ T cells

**Figure 6:**
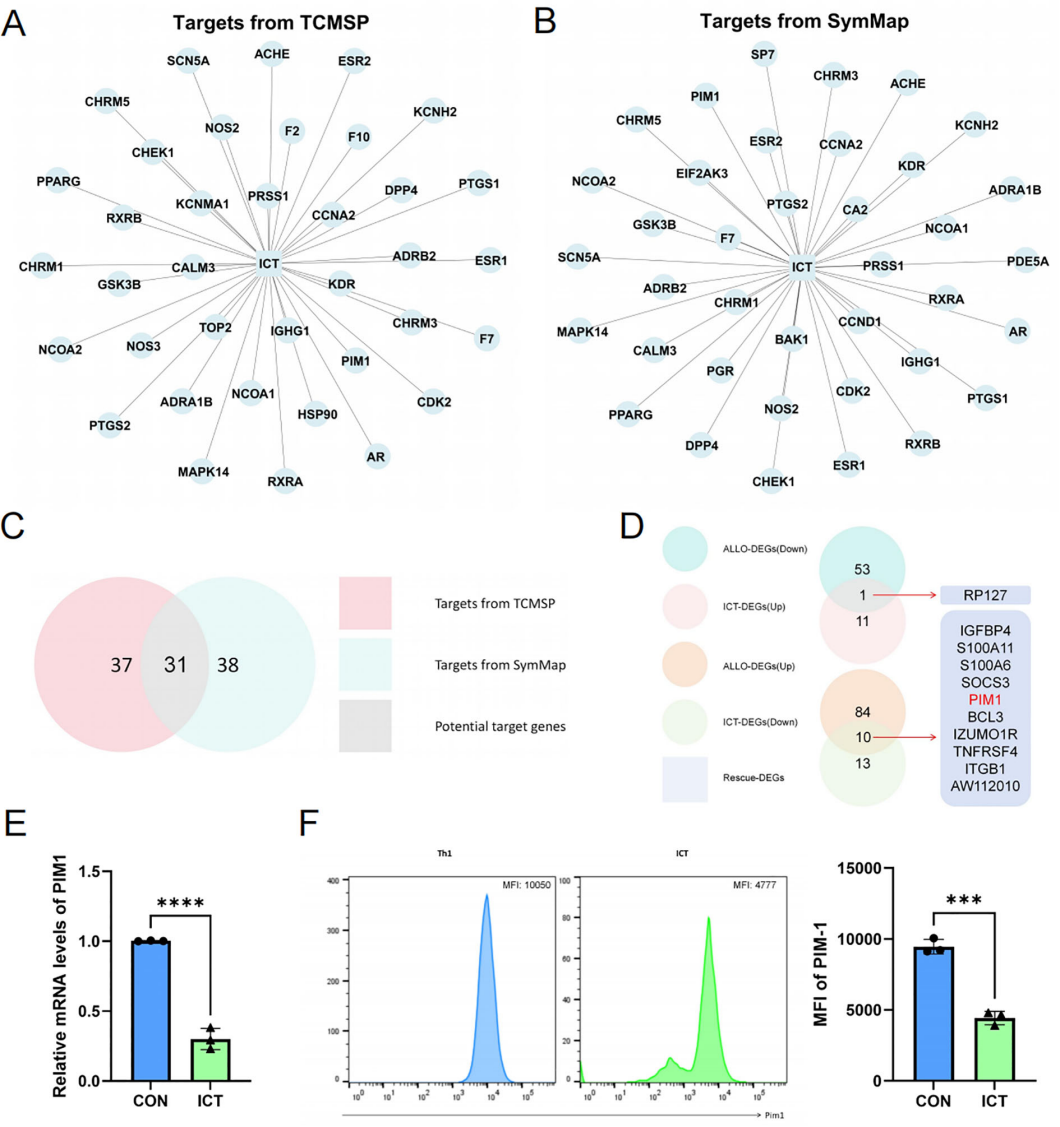
ICT inhibits PIM1 expression in CD4^+^ T cells. **(A)** Targets of ICT predicted by the TCMSP database. **(B)** Targets of ICT predicted by the SymMap database. **(C)** Venn diagram showing the potential target genes associated with ICT. **(D)** Venn diagram illustrating the Rescue-DEGs of CD4^+^ T cells. **(E)** mRNA expression of PIM1 in ICT-treated CD4^+^ T cells. **(F)** Protein expression of PIM1 in ICT-treated CD4^+^ T cells.

To further explore how ICT inhibits CD4^+^ T cell activation, proliferation and differentiation, we used the Traditional Chinese Medicine Systems Pharmacology Database and Analysis Platform (TCMSP) (**Figure 6A**) and the SymMap database (**Figure 6B**) to integrate and screen the potential targets of ICT. As shown in **Figure 6C**, 31 potential target genes associated with ICT were identified. Next, from the scRNA-seq data of CD4^+^ T cells, we identified 11 Rescue-DEGs by comparing the upregulated ALLO-DEGs with the downregulated ICT-DEGs, and the downregulated ALLO-DEGs with the upregulated ICT-DEGs, respectively (**Figure 6D**). The only overlapping gene between the Rescue-DEGs and the potential target genes was PIM1, a member of the serine/threonine protein kinase family. Previous studies have demonstrated that PIM1 inhibition suppresses CD4^+^ T-cell activation, proliferation, and Th1 differentiation (22, 23). Therefore, we hypothesize that ICT exerts immunosuppressive effects via suppression of PIM1 in CD4^+^ T cells. *In vitro*, CD4^+^ T cells were treated with ICT at a concentration of 4 µM, which significantly inhibited Th1 cell differentiation. As shown in **Figures 6E, F**, ICT significantly reduced PIM1 mRNA expression, as determined via RT-qPCR and protein expression, as determined via by flow cytometry. These results indicate that ICT significantly inhibits the expression of PIM1, a kinase that regulates the activation, proliferation, and Th1 differentiation of CD4^+^ T cells.

### 3.7 ICT inhibits Th1 cell differentiation by directly binding the transcription factor CEBPB

**Figure 7:**
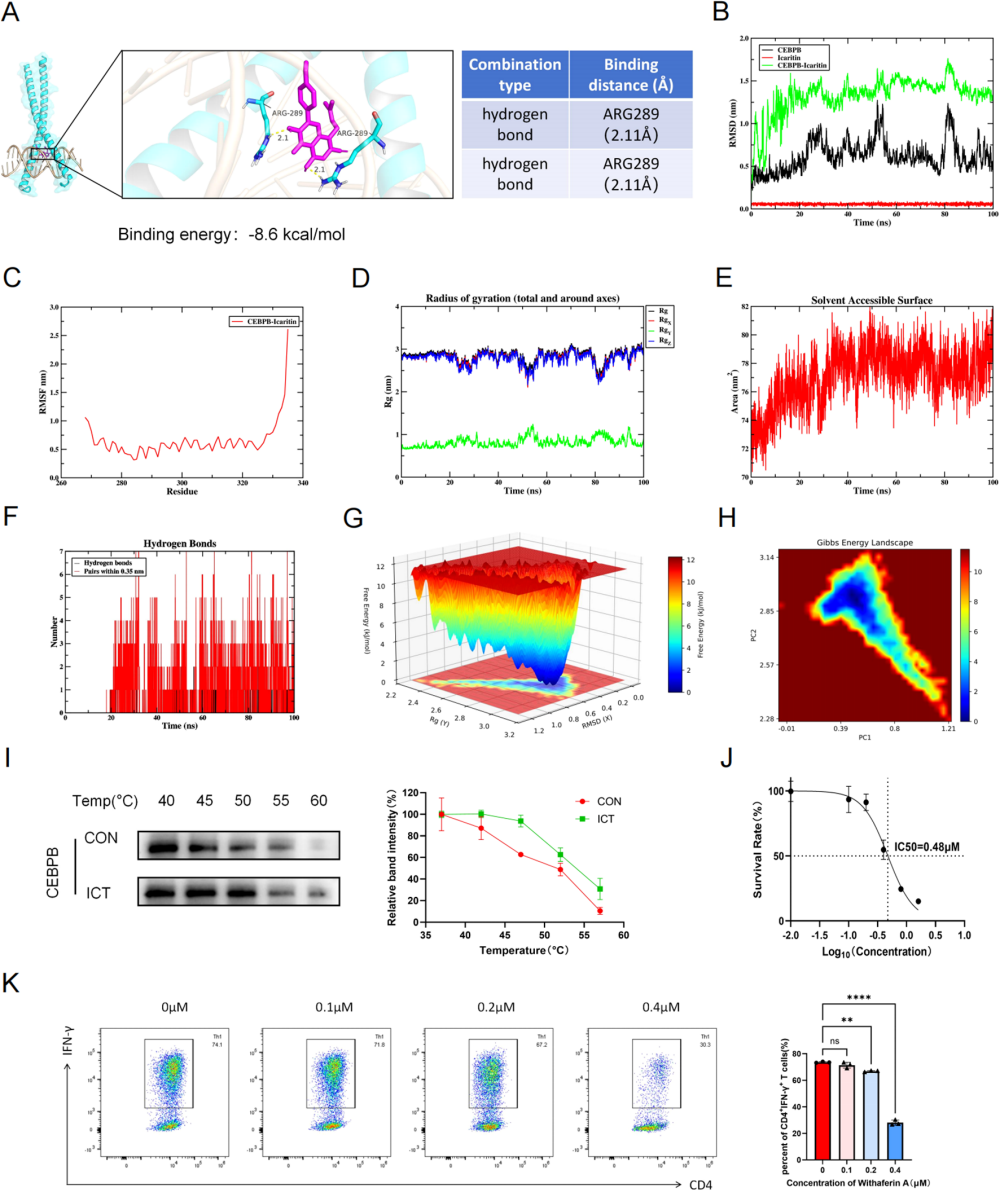
ICT binds to CEBPB and inhibits Th1 differentiation in CD4^+^ T cells. **(A)** CEBPB-ICT binding interaction predicted by molecular docking. **(B-H)** Molecular dynamics simulations analysis of CEBPB-ICT. **(B)** The RMSD plot of CEBPB-ICT. **(C)** The RMSF plot of CEBPB-ICT. **(D)** The Rg plot of CEBPB-ICT. **(E)** The SASA plot of CEBPB-ICT. **(F)** Number of hydrogen bonds between CEBPB and ICT. **(G)** 3D FEL plots of CEBPB-ICT. **(H)** 2D FEL plots of CEBPB-ICT. **(I)** CETSA validation of CEBPB-ICT target engagement in CD4^+^ T cells. **(J)** IC_50_ of withaferin A on CD4^+^ T cells was identified. **(K)** Percentages of CD4^+^ IFN-γ^+^ Th1 cells treated with different concentration of withaferin **(A)** Statistical analysis was conducted by one-way ANOVA more than three groups, and unpaired t test was used to compared two groups. Data are shown as mean ± SD; ns, *p* > 0.05, **p < 0.01, ****p < 0.0001.

Following the validation that PIM1 is the target of ICT in CD4^+^ T cells, further analysis was performed to determine the mechanism through which ICT targets PIM1 and inhibits Th1 cell differentiation. CEBPB, a key member of the b-ZIP transcription factor family, has been demonstrated to specifically bind to the promoter of PIM1 and enhance its transcriptional activation (24). CEBPB-deficient mice exhibit impaired IFN-γ production capacity in macrophages, dendritic cells, and CD4^+^ T cells (29). Therefore, we hypothesize that ICT regulates PIM1 transcription by targeting CEBPB, thereby further inhibiting Th1 cell differentiation. Herein, molecular docking was employed to explore the interaction between ICT and CEBPB. As shown in **Figure 7A**, ICT strongly interacts with the CEBPB protein with a binding energy of −8.6  kcal/mol. Specifically, hydrogen bonds were formed with residue ARG289 of chain A (2.11 Å) and residue ARG289 of chain B (2.11 Å) in the CEBPB dimer. Furthermore, we select ICT and CEBPB for molecular dynamics simulation. As shown in **Figure 7B**, the complex stabilized after 50 ns of simulation, with its RMSD fluctuating around a stable value of 1.4 nm, which suggests a highly stable binding mode between ICT and CEBPB. The low RMSF values (mostly < 0.6 nm) of the complex indicate its low flexibility and high stability (**Figure 7C**). As shown in **Figure 7D**, during the simulation, the Rg of the complex exhibited minor fluctuations, indicating that the overall compactness of the structure was maintained. As a metric for evaluating protein surface area, SASA did not alter significantly after the formation of the ICT-CEBPB complex, implying that the binding of ICT induces little structural change in CEBPB (**Figure 7E**). As shown in **Figure 7F**, the number of hydrogen bonds between ICT and CEBPB ranges from 0--7, indicating favorable hydrogen bonding interactions between them. To comprehensively investigate the dynamic conformational stability and intrinsic interactions of the complex system, we constructed 2D and 3D FEL plots. The results revealed a global free energy minimum region (represented by dark blue basins in the plot) within specific ranges of Rg (2.92 nm) and RMSD (0.60 nm), indicating that the system reaches its thermodynamically most stable state in this conformational ensemble (**Figures 7G, H**). To validate the direct binding of ICT and CEBPB, we performed a CETSA to assess target protein thermal stability following compound binding. The results showed that ICT inhibited heat-induced degradation of CEBPB (**Figure 7I**). To confirm that ICT regulates Th1 cell differentiation by targeting CEBPB, we introduced Withaferin A, a highly selective inhibitor of CEBPB (30), to assess its potential to inhibit Th1 cell differentiation. We determined that withaferin A had an IC_50_ of 0.48 µM for CD4^+^ T cells (**Figure 7J**). As shown in **Figure 7K**, withaferin A inhibited Th1 cell differentiation in a dose-dependent manner *in vitro*. Taken together, these findings indicate that ICT directly binds to CEBPB, the transcription factor of PIM1, thereby inhibiting Th1 cell differentiation.

### 3.8 ICT potentiated the immunosuppressive efficacy of tacrolimus while reducing the HCC burden

**Figure 8:**
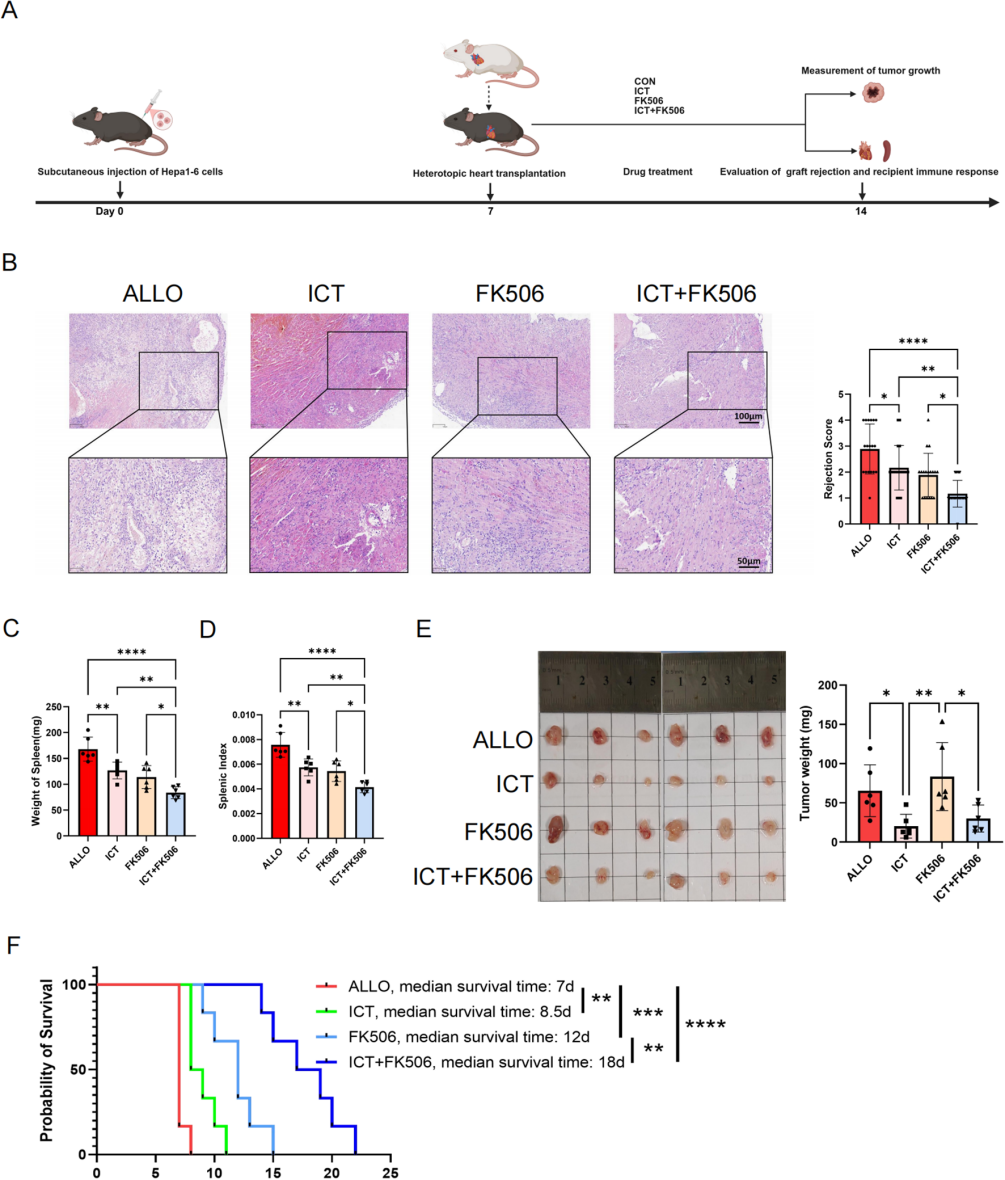
The combination of ICT and tacrolimus attenuates acute allograft rejection while suppressing tumor growth in mice. **(A)** Schematic diagram demonstrating the establishment of the HCC-bearing heterotopic heart transplant model and assessment of allograft rejection and tumor growth. **(B)** Allograft histopathological changes were evaluated by H&E staining across groups. Acute rejection severity was graded based on cardiac rejection scoring criteria, with 3 randomly selected areas analyzed per slide. **(C)** The spleen weights of the recipients. **(D)** The spleen indices of the recipients. **(E)** The weights of the tumors. **(F)** Kaplan - Meier curves of cardiac allograft survival. Statistical analysis was conducted by one-way ANOVA (n = 6 mice per group). Data are shown as mean ± SD; ns, *p* > 0.05, *p < 0.05, **p < 0.01, ***p < 0.001, ****p < 0.0001.

Given that ICT attenuates acute allograft rejection by suppressing CD4^+^ T cell activation, proliferation, and Th1 differentiation, we further employed a HCC-bearing murine heart transplantation model to investigate its potential dual efficacy in simultaneously attenuating allograft rejection and exerting antitumor effects. Recipient mice were stratified into 4 groups and administered: vehicle control (ALLO), ICT, tacrolimus (FK506) and a combination of ICT + tacrolimus (ICT+FK506) (**Figure 8A**). As depicted in **Figure 8B**, the ALLO group exhibited marked infiltration of inflammatory cells within the graft, accompanied by hemorrhage and degradation of the cardiac architecture. Monotherapy with either ICT or tacrolimus markedly reduced inflammatory cell infiltration compared to the untreated group. Remarkably, the ICT + tacrolimus combination demonstrated superior immunosuppressive capabilities, dramatically curtailing acute allograft rejection and decreasing the allograft rejection score. In addition, the spleen weight (**Figure 8C**) and splenic index (**Figure 8D**) were markedly decreased in the combination group, further indicating that ICT + tacrolimus cotreatment markedly inhibited alloantigen-driven splenocyte proliferation. Impressively, the HCC burden in ICT and ICT + tacrolimus-treated recipient mice was significantly lower than that in the FK506 group (**Figure 8E**). As shown in **Figure 8F**, the combination of ICT and tacrolimus significantly prolonged the graft survival time from 7 d in control mice to 17 d. Taken together, ICT concurrently attenuated acute allograft rejection and reduced HCC burden in recipients.

## 4 Discussion

There is a dual risk of both rejection and malignancy after organ transplantation, particularly in patients undergoing liver transplantation for HCC. The long-term immunosuppressive status required to prevent rejection significantly increases the risk of malignancy. While cancer immunotherapies have shown great efficacy and promise, its use in transplant recipients has been linked to a higher incidence of rejection (31). Therefore, developing a strategy capable of simultaneously attenuating allograft rejection and reducing the risk of malignancy in the context of transplantation is imperative. RAPA is a well-recognized immunosuppressant with antitumor properties (32), and it has been widely used in LT for HCC (33, 34). However, the early use of RAPA was limited by side effects such as HAT and impaired wound healing (35, 36). Consequently, there is an imperative need to develop novel immunosuppressants with antitumor properties.

While clinically used against HCC, ICT has been reported to exert anti-inflammatory effects in various autoimmune diseases (14–16), representing a potential immunosuppressant with antitumor properties. A clinical trial investigating ICT for advanced HCC observed only grade I/II adverse reactions, with no grade III/IV treatment-related adverse reactions (12). This suggests that ICT has a better safety profile compared to rapamycin. However, the potential therapeutic roles and mechanisms of ICT in attenuating allograft rejection remain unclear.

In this study, we demonstrated through an allogeneic mouse heart transplantation model that ICT significantly attenuated cardiac allograft rejection and prolonged graft survival. To explore the underlying mechanisms of ICT therapy in allograft rejection, we performed scRNA-seq on recipient spleens. GO analysis showed that ICT decreased the biological processes related to T cell activation, cytokine pathways, and T cell differentiation. In allograft rejection, T cells are the primary immune cells responsible for graft damage (18). Currently, most immunosuppressants commonly used in clinical organ transplantation exert their effects by directly or indirectly inhibiting T-cell activity or function (37). To better evaluate the therapeutic effects of ICT on acute allograft rejection, we further analyzed the changes in T cell subsets. GSEA analysis of CD4^+^ T cells indicated that ICT inhibits CD4^+^ T-cell differentiation into Th1 cells, which have traditionally been considered the main participants in rejection (38). Flow cytometry confirmed that ICT significantly reduced the proportion of Th1 cells among CD4^+^ T cells in the spleens of recipient mice. In addition, the serum levels of the Th1 cytokine IFN-γ were significantly lower in the ICT group than in the ALLO group, further supporting that ICT inhibits the differentiation of CD4^+^ T cells into Th1 cells *in vivo*. To confirm the direct effect of ICT on CD4^+^ T cells, we conducted *in vitro* experiments and demonstrated that ICT inhibits CD4^+^ T-cell activation, proliferation and Th1 differentiation in a dose-dependent manner. These results suggest that ICT alleviates acute allograft rejection by inhibiting CD4^+^ T-cell activation, proliferation and Th1 differentiation.

To investigate the mechanism by which ICT inhibits CD4^+^ T-cell activation, proliferation and Th1 differentiation thereby attenuating acute rejection, we predicted potential targets of ICT via network pharmacology and analyzed Rescue-DEGs through scRNA-seq. The intersection of these analyses identified PIM1 as a key candidate gene. PIM1 is a serine/threonine kinase that is recognized as a proto-oncogene in the field of oncology (39). In recent years, research has focused on its role in immunomodulation. The aberrant activation of PIM1 in CD4^+^ T cells is associated with various autoimmune diseases, such as autoimmune uveitis and inflammatory bowel disease, and may serve as a potential therapeutic target (19). It has been reported that PIM1 is required for the activation, proliferation, and differentiation of CD4^+^ T cells (22, 23). Next, we confirmed that ICT can suppress the expression of PIM1 at both the RNA and protein levels, suggesting that PIM1 may be a key factor through which ICT suppresses CD4^+^ T-cell activation, proliferation, and Th1 cell differentiation. Notably, PIM1 serves dual roles as both a proto-oncogene that drives tumorigenesis and a key immune regulator involved in inflammatory signal transduction (19, 20). This unique dual functionality positions PIM1 as a promising therapeutic target for the development of novel agents that concurrently exert anti-tumor and immunomodulatory effects. CEBPB acts as a transcriptional regulator of the PIM1 gene, directly binding to the PIM1 promoter region and upregulating the expression of the NLRP3 inflammasome along with the proinflammatory cytokines IL-1β and IL-6 (24). CEBPB-deficiency has been demonstrated to impair IFN-γ production in macrophages, dendritic cells, and CD4^+^ T cells (29). Thus, we confirmed that ICT robustly interacts with CEBPB, and that CEBPB inhibitors effectively suppress Th1 cell differentiation *in vitro*. These results reveal that ICT inhibits Th1 cell differentiation through the CEBPB/PIM1 pathway.

In clinical post-transplant management, combination immunosuppressive regimens are routinely employed to achieve adequate immunosuppression. Herein, we assessed the immunosuppressive efficacy and impact on tumor burden of ICT monotherapy, tacrolimus monotherapy, and ICT-tacrolimus combination therapy in the tumor-bearing murine cardiac allograft model. The ICT-tacrolimus combination regimen induced long-term graft survival and simultaneously reduced the HCC burden. These results substantiate the inclusion of ICT as a combinatorial agent in posttransplant immunosuppressive regimens, augmenting therapeutic efficacy while suppressing the risk of malignancy.

A limitation of this study is that although ICT monotherapy significantly prolonged graft survival, it failed to induce long-term graft acceptance. The primary clinical value of ICT lies in its combination with potent immunosuppressive regimens, concurrently augmenting graft protection and mitigating malignancy risk. Furthermore, the potential interactions between ICT and other first-line immunosuppressants in the context of organ transplantation remain undefined and warrant further investigation.

In summary, we demonstrated that ICT attenuates allograft rejection through hindering CD4^+^ T-cell activation, proliferation and Th1 differentiation, which is mediated by inhibition of the CEBPB/PIM1 pathway. ICT represents a potential immunosuppressant with antitumor activity for clinical organ transplantation, particularly in liver transplantation for HCC.

## Data Availability

The raw single-cell RNA sequencing data generated in this study have been deposited in the NCBI Sequence Read Archive (SRA) under the BioProject accession number PRJNA1377001. The target prediction for ICT was performed using the following public databases: the TCMSP (https://www.tcmsp-e.com/) and the SymMap database (http://www.symmap.org/). Further inquiries can be directed to the corresponding authors.
