# Supplementary material for: Dual role of icaritin in attenuating allograft rejection and exerting antitumor effects in mice
Source: Front Immunol. 2026 Mar 18;17:1762553. doi: 10.3389/fimmu.2026.1762553 (PMC13038598; doi:10.3389/fimmu.2026.1762553)
Supplement: Supplementary file 1 [file DataSheet1.docx]

**
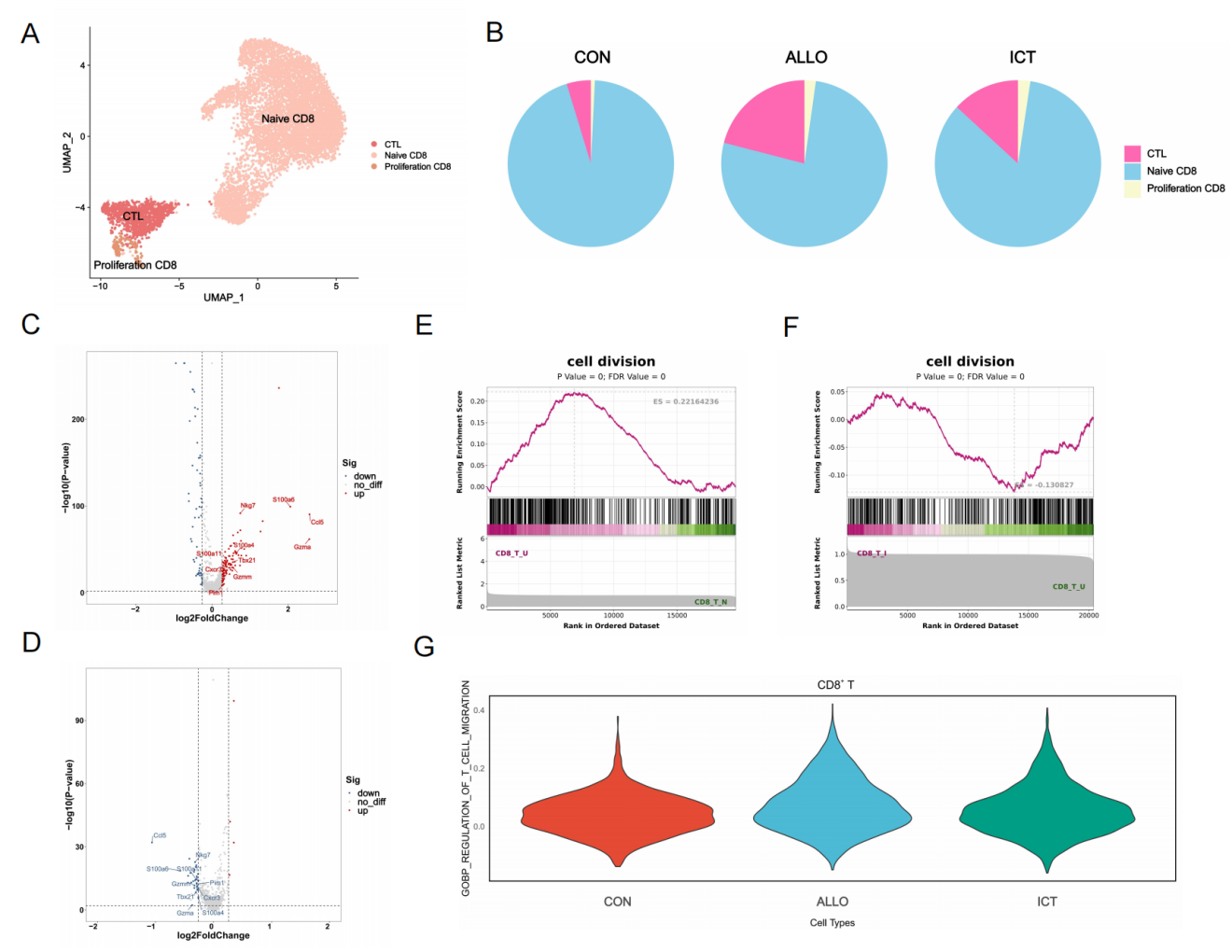
Fig. S1. scRNA-seq analysis of the ICT mediated response in CD4^+^ T cells**

(A) UMAP plots displaying subclusters of CD8^+^ T cells. (B) Pie chart showing the proportional distribution of CD8^+^ T cell subtypes. (C) Volcano plot showing the upregulated ALLO-DEGs in CD8^+^ T cells. (D) Volcano plot showing the downregulated ICT-DEGs in CD8^+^ T cells. (E) GSEA analysis of upregulated ALLO-DEGs enriched in KEGG in CD8^+^ T cells. (F) GSEA analysis of downregulated ICT-DEGs enriched in KEGG in CD8^+^ T cells. (G) Violin plot showing the score of regulation of T cell migration pathway in CD8^+^ T cells among three groups.
