# Supplementary material for: Dual role of icaritin in attenuating allograft rejection and exerting antitumor effects in mice
Source: Front Immunol. 2026 Mar 18;17:1762553. doi: 10.3389/fimmu.2026.1762553 (PMC13038598; doi:10.3389/fimmu.2026.1762553)
Supplement: Supplementary file 2 [file DataSheet2.docx]

**
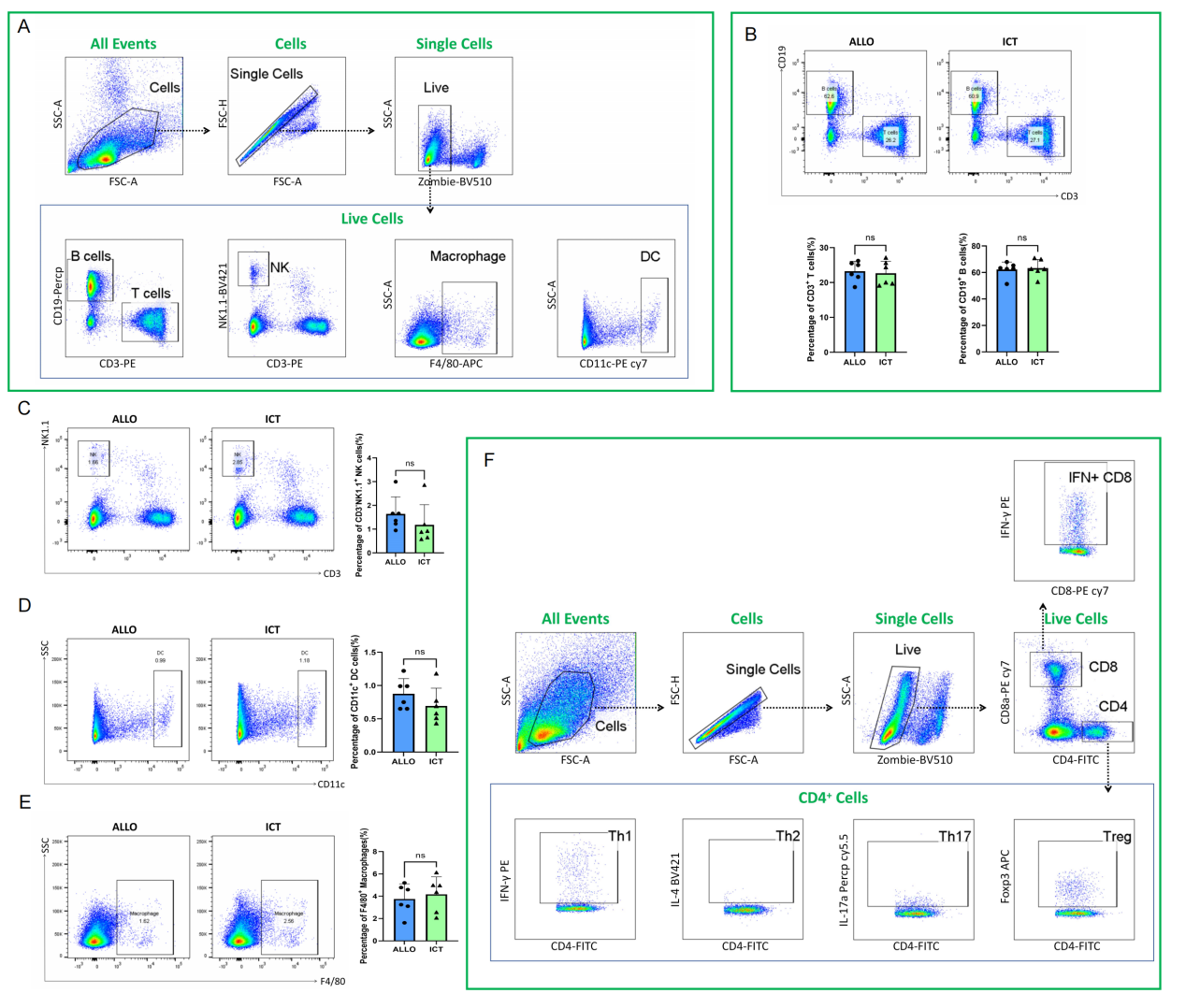
Fig. S2. Flow cytometry analysis of the proportions of various immune cell types**

(A) Gating strategy for flow cytometric analysis of T, B, NK cells, dendritic cells, and macrophages. (B) Flow cytometry analysis of the percentages of CD3^+^ CD19B^-^ T and CD3^-^ CD19B^+^ B cells. (C) Flow cytometry analysis of the percentage of NK1.1^+^ NK cells. (D) Flow cytometry analysis of the percentage of CD11c^+^ dendritic cells. (E) Flow cytometry analysis of the percentage of F4/80^+^ macrophages. (F) Gating strategy for flow cytometric analysis of Th1, Th2, Th17 and Treg cells. Statistical analysis between two groups was conducted by Student’s t-test (n = 6 mice per group). Data are shown as mean ± SD; ns, *p* > 0.05, * *p* < 0.05, ***p* < 0.01, ****p* < 0.001, *****p* < 0.0001.
