# Supplementary material for: Dual role of icaritin in attenuating allograft rejection and exerting antitumor effects in mice
Source: Front Immunol. 2026 Mar 18;17:1762553. doi: 10.3389/fimmu.2026.1762553 (PMC13038598; doi:10.3389/fimmu.2026.1762553)
Supplement: Supplementary file 3 [file DataSheet3.docx]

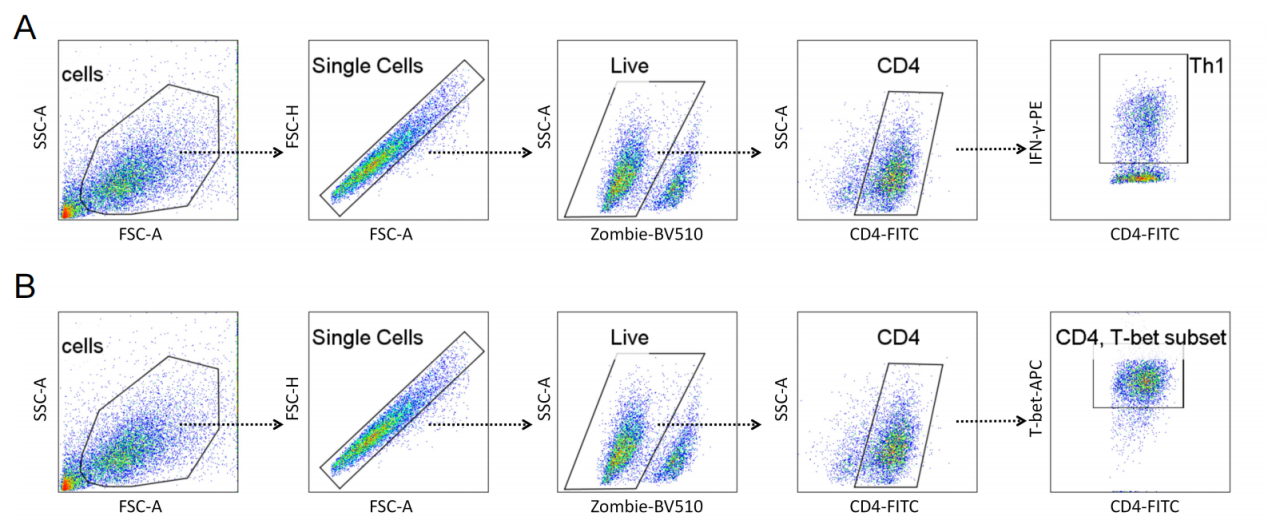


**Fig. S3. Flow cytometry analysis of the proportions of Th1 cells**

(A) Gating strategy for flow cytometric analysis of CD4^+^ IFN-γ^+^ cells. (B) Gating strategy for flow cytometric analysis of CD4^+^ T-bet^+^ cells.
